# The complete chloroplast genome of *Ziziphus jujuba* cv. Bokjo (Rhamnaceae)

**DOI:** 10.1080/23802359.2022.2131366

**Published:** 2022-10-17

**Authors:** Moonkyo Kim, Jin Hee Park, Jinsu Gil, Jung Sung Kim, Hyoung Tae Kim, Ha Kyung Oh, Kyeong Hee Lee, Minjee Lee, Jungho Lee, Yi Lee

**Affiliations:** a Department of Industrial Plant Science & Technology, Chungbuk National University, Cheongju, Republic of Korea; b Korea Polar Research Institute, Incheon, Republic of Korea; c Nakdonggang National Institute of Biological Resources, Sangju, Republic of Korea; d Department of Forest Science, Chungbuk National University, Cheongju, Republic of Korea; e College of Ecology and Environmental Science, Kyungpook National University, Sangju, Republic of Korea; f Chungcheongbuk-do Agricultural Research & Extension Services, Cheongju, Republic of Korea; g Green Plant Institute, Yongin, Republic of Korea

**Keywords:** Chloroplast genome, next-generation sequencing, *Ziziphus jujuba*

## Abstract

We have sequenced the *Ziziphus jujuba* cv. Bokjo chloroplast genome by *de novo* assembly using next-generation sequencing. The complete circular chloroplast genome consisted of 161,714 bp and contained four parts: a large single-copy (LSC) region of 89,323 bp, a small single-copy (SSC) region of 19,361 bp, and two inverted repeat regions (IRa and IRb) of 26,515 bp each. The genome annotation predicted a total of 110 genes, including 76 protein-coding genes, 30 tRNA genes, and four rRNA genes. Phylogenetic analysis demonstrated the close taxonomic relationship between *Z. jujuba* cv. Bokjo and two other members of the *Ziziphus* genus, *Z. spina-christi* and *Z. mauritiana*. We found 135 polymorphic loci, 63 single nucleotide polymorphism (SNP) and 72 insertion–deletion (InDel), from the comparison of *Z. jujuba* cultivar Bokjo and *Z. jujuba* reference (NC_030299). The polymorphic loci could be used for the differentiation of *Z. jujuba* genetic resources and for breeding in the future.

Jujube (*Ziziphus jujuba* Mill. 1768) is a deciduous tree belonging to the genus *Ziziphus* of the Rhamnaceae family. *Z. jujuba* inhabits subtropical and tropical arid regions, but some species occur in temperate and arid regions with continental climates (Evreinoff 1964; Nam et al. 2015). *Z. jujuba* extract contains the alkaloids lysicamine and nornuciferine and is an effective sedative (Han and Park 1987). A long period of evolution and artificial selection has given rise to various varieties and cultivars of jujube, and more than 800 cultivars have been reported (Wang et al. 2014). Despite the economic importance of jujube, molecular research on the species is limited, as is knowledge concerning its genetic diversity and cultivar identification (Liang et al. 2019).

We have completed the chloroplast genome of *Z. jujuba* cv. Bokjo, the most widely cultivated jujube cultivar in Korea (Lee et al. 2018). *Z. jujuba* cv. Bokjo leaf samples for DNA extraction were collected from Chungcheongbuk-do Agricultural Research and Extension Service (CBARES) (36°34′38.7″N, 127°44′52.9″E). A dried plant specimen was deposited in the Herbarium of the National Institute of Horticulture and Herbal Science, Eumsung, Republic of Korea (http://www.nihhs.go.kr/; Contact: Yoongee Lee; yoong0625@korea.kr) under the voucher number MPS006272. Total genomic DNA was extracted using the DNeasy Plant Mini Kit (Qiagen, Valencia, CA). The isolated genomic DNA was used to construct a paired-end (PE) library with a mean insert size of 700 bp, using the Illumina HiSeq platform. Contigs were assembled with a total of 11.96 Gbp PE reads collected from Illumina Hiseq next-generation sequencing using the CLC Genomics Workbench (ver. 11.0, Qiagen, Aarhus, Denmark). The assembled structure and the genes of the complete chloroplast genome were annotated using the DOGMA program (http://dogma.ccbb.utexas.edu/) (Wyman et al. 2004) and were manually corrected based on a BLAST search. The *Z. jujuba* cv. Bokjo complete chloroplast genome sequence was deposited in GenBank under accession no. MT919946. The complete *Z. jujuba* cv. Bokjo chloroplast genome was a circular molecule with a length of 161,714 bp and a GC content of 36.75%, and was composed of four distinct regions: a large single-copy region of 89,323 bp, a small single-copy region of 19,361 bp, and two inverted repeat regions (IRa and IRb) each of 26,515 bp. We annotated 110 genes within the *Z. jujuba* cv. Bokjo chloroplast genome, which included 76 protein-coding genes, 30 tRNA genes, and four rRNA genes.

We performed a phylogenetic analysis based on the complete chloroplast genome sequence of *Z. jujuba* cv. Bokjo and of 11 other species, including five ingroup species of the Rhamnaceae to which *Ziziphus* belongs, and six outgroup species of the Rosales. To generate the phylogenetic tree, we used the time-reversal model of the maximum-likelihood algorithm with 1000 bootstrap replications in the CLC Genomics Workbench (ver. 11.0, CLC Qiagen). Six taxa of Rhamnaceae formed a monophyletic group that was distinct from six other species of the Rosales (Figure 1). The phylogenetic tree placed *Z. jujuba* cv. Bokjo together with *Z. jujuba* (NC_030299) and close to *Z. spina-christi* and *Z. mauritiana*.

**Figure 1.**
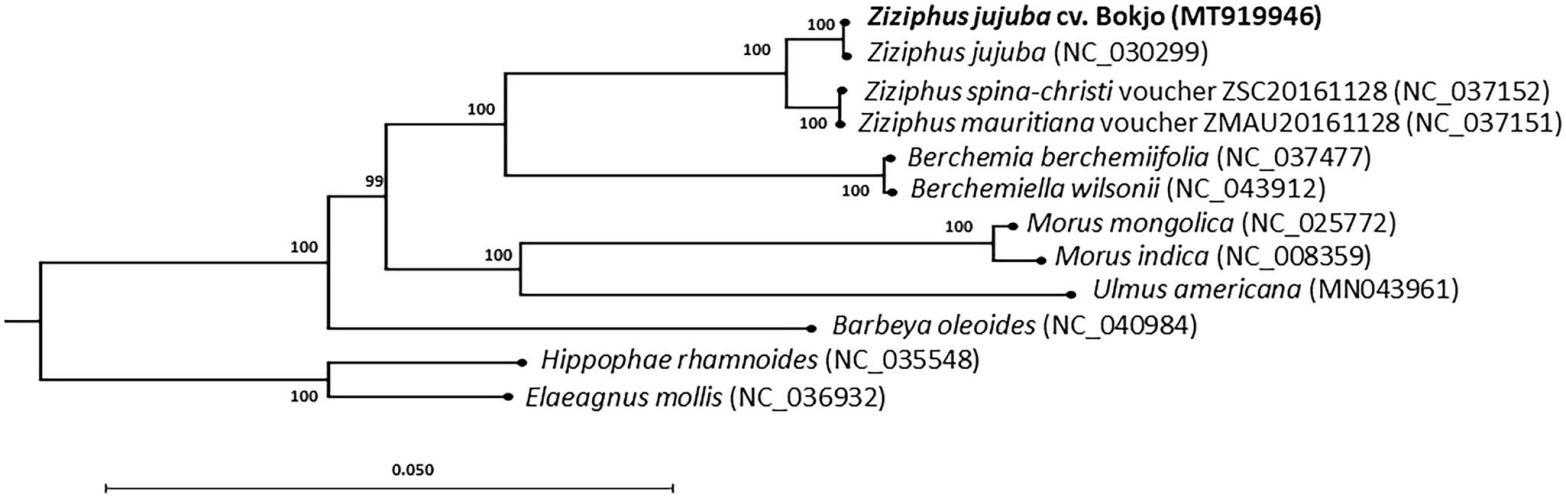
Maximum-likelihood phylogenetic tree based on the chloroplast genome sequence from *Z. jujuba* cv. Bokjo and related species in the Rhamnaceae family, to which the genus *Ziziphus* belongs. The chloroplast sequences of members of the Elaeagnaceae, Barbeyaceae, Moraceae, and Ulmaceae families were used as the outgroup. Numbers at each node represent the bootstrap values for 1000 replicates.

We compared the chloroplast genome of *Z. jujuba* cultivar Bokjo to a *Z. jujuba* reference (NC_030299) and we found 135 polymorphic loci, 63 single nucleotide polymorphisms (SNPs) and 72 InDels. The polymorphic loci could be used for the differentiation of *Z. jujuba* genetic resources and for breeding in the future.

## Data Availability

The genome sequence data that support the findings of this study are openly available in GenBank of NCBI at https://www.ncbi.nlm.nih.gov under the accession no. MT919946. The associated ‘BioProject’, ‘SRA’, and ‘Bio-Sample’ numbers are PRJNA794271, SRR17438409, and SAMN24619238, respectively.
